# A new species of the genus *Xizangiana* Sherwood, Li & Zhang, 2022 (Araneae, Gnaphosidae) from Xizang, China

**DOI:** 10.3897/BDJ.12.e116569

**Published:** 2024-01-19

**Authors:** Bo Liu, Feng Zhang

**Affiliations:** 1 Hebei University, Baoding, China Hebei University Baoding China

**Keywords:** ground spider, Tibet, taxonomy, morphology

## Abstract

**Background:**

The spider genus *Xizangiana* Sherwood, Li & Zhang, 2022, comprises nine species that inhabit northern India and south-western China. Six of these species have been documented in the Xizang Autonomous Region of China.

**New information:**

A new species, *Xizangianaplankton* sp. nov. is described and illustrated from Xizang Autonomous Region, China.

## Introduction

Gnaphosidae is a highly diverse spider family, containing 2468 extant described species in 151 genera (WSC 2023). *Xizangiana* Sherwood, Li & Zhang, 2022 is a small genus of Gnaphosidae, with nine species occurring in northern India and south-western China, and six of them are reported in Xizang Autonomous Region, China (Liu et al. 2023, WSC 2023).

Liu et al. (2023) revised *Xizangiana* Sherwood, Li & Zhang, 2022 and identified its species diversity in Xizang. However, the current knowledge of *Xizangiana* species in this region is inadequate. In 2023, during a spider expedition in Rigaze City, Xizang Autonomous Region, the 10^th^ species of *Xizangiana* was discovered, and named *Xizangianaplankton* sp. nov.

## Materials and methods

All specimens preserved in 75% ethanol were examined and measured under a Leica M205A stereomicroscope. Photographs were taken using an Olympus BX51 microscope, equipped with a Kuy Nice CCD camera and were imported into Helicon Focus v. 7 for stacking. Final figures were retouched by the Adobe Photoshop © 2020. All measurements are given in millimeters. Leg measurements are shown as: total length (femur, patella, tibia, metatarsus, tarsus). Female genitalia were cleared with Pancreatin (BBI Life Sciences). All specimens studied are deposited in the Museum of Hebei University (MHBU), Baoding, China.

Abbreviations used in this study: **AF**, anterior fold; **ALE**, anterior lateral eyes; **AME**, anterior median eyes; **BG**, Bennett’s gland; **CD**, copulatory duct; **E**, embolus; **EB**, embolar base; **EBP**, embolar base projection; **EP**, embolar process; **FD**, fertilisation ducts; **LF**, lateral fold; **MA**, median apophysis; **MP**, membranous projection; **Pi**, piriform gland spigots; **PS**, primary spermathecae; **PLE**, posterior lateral eyes; **PME**, posterior median eyes; **R**, embolar radix; **SC**, scape; **SD**, sperm duct; **SS**, secondary spermathecae; **ST**, subtegulum; **T**, tegulum; **TF**, transversal folds.

## Taxon treatments

### Xizangiana plankton

Liu & Zhang sp. nov.

88BED3E8-FB20-5951-9E09-1917ECDD6BC1

9CF3A7E5-F5D0-48B7-A71E-7E48872B40BF

#### Materials

- **Type status:** Holotype. **Occurrence:** recordedBy: Bo Liu; individualCount: 1; sex: male; lifeStage: adult; occurrenceID: 970D06DF-F671-5D97-9060-6EB2A16F2915; **Taxon:** scientificName: Xizangianaplankton; **Location:** country: China; stateProvince: Xizang; county: Renbu; locality: Padang Town; verbatimElevation: 3856 m; verbatimCoordinates: 30°20.56'N 119°26.03'E; decimalLatitude: 29.3137; decimalLongitude: 90.2677; **Identification:** identifiedBy: Bo Liu; dateIdentified: 2023; **Event:** year: 2023; month: 8; day: 12; **Record Level:** institutionID: the Museum of Hebei University; institutionCode: MHBU-ARA-2023-906-1
- **Type status:** Paratype. **Occurrence:** recordedBy: Bo Liu; individualCount: 1; sex: female; lifeStage: adult; occurrenceID: F4ECB603-04DB-5BA6-B3A9-6DFD8D11C46B; **Taxon:** scientificName: Xizangianaplankton; **Location:** country: China; stateProvince: Xizang; county: Renbu; locality: Padang Town; verbatimElevation: 3856 m; verbatimCoordinates: 30°20.56'N 119°26.03'E; decimalLatitude: 29.3137; decimalLongitude: 90.2677; **Identification:** identifiedBy: Bo Liu; dateIdentified: 2023; **Event:** year: 2023; month: 8; day: 12; **Record Level:** institutionID: the Museum of Hebei University; institutionCode: MHBU-ARA-2023-906-2
- **Type status:** Paratype. **Occurrence:** recordedBy: Bo Liu; individualCount: 1; sex: male; lifeStage: adult; occurrenceID: F1A51DD7-D1E0-5429-B8E5-8AEA19D46968; **Taxon:** scientificName: Xizangianaplankton; **Location:** country: China; stateProvince: Xizang; county: Renbu; locality: Padang Town; verbatimElevation: 3856 m; decimalLatitude: 29.3137; decimalLongitude: 90.2677; **Identification:** identifiedBy: Bo Liu; dateIdentified: 2023; **Event:** year: 2023; month: 8; day: 12; **Record Level:** institutionID: the Museum of Hebei University; institutionCode: MHBU-ARA-2023-906-3

#### Description

**Figure 1.**
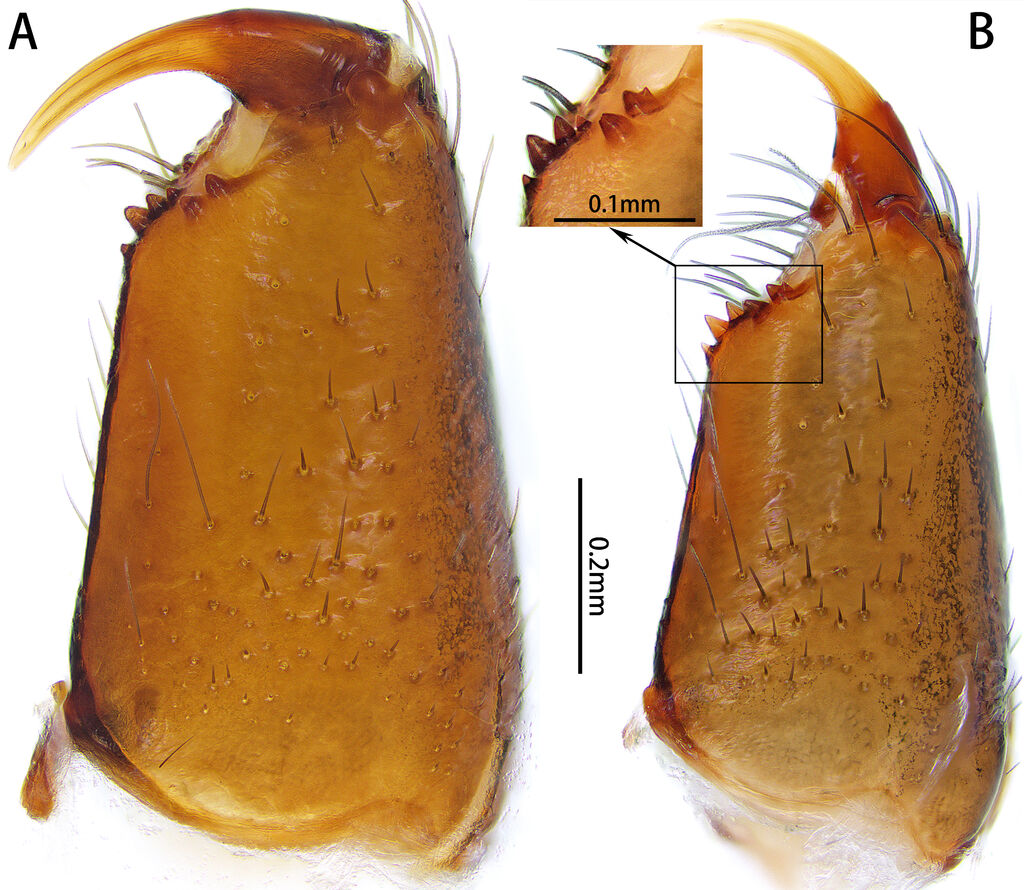
Left chelicerae of *Xizangianaplankton* sp. nov. **A** female, retrolateral view; **B** male, retrolateral view.

Male. Total length 3.38–3.85. Holotype: total length 3.38; carapace 1.87 long, 1.43 wide; abdomen 1.51 long, 1.16 wide. Eye sizes and interdistances: AME 0.06, ALE 0.10, PME 0.07, PLE 0.08; AME‒AME 0.05, AME‒ALE 0.01, PME‒PME 0.05, PME‒PLE 0.05, ALE‒PLE 0.05. Leg measurements: I 5.08 (1.48, 0.65, 1.32, 0.87, 0.76), II 4.14 (1.20, 0.56, 0.94, 0.76, 0.68), III 3.72 (1.08, 0.45, 0.74, 0.82, 0.63), IV 5.95 (1.60, 0.74, 1.25, 1.51, 0.85). Cheliceral promargin with 4 teeth, retromargin with 2 (Fig. 1B). Anterior lateral spinnerets with 4 enlarged piriform gland spigots (Fig. 3C). Colour in alcohol (Fig. 3A‒B): carapace dark brown, legs yellow-brown. Abdomen black-grey with 2 large white markings anterolaterally, 2 small white markings medially, several chevron-like white stripes posteriorly and two longitudinal black stripes ventrally.

**Figure 2.**
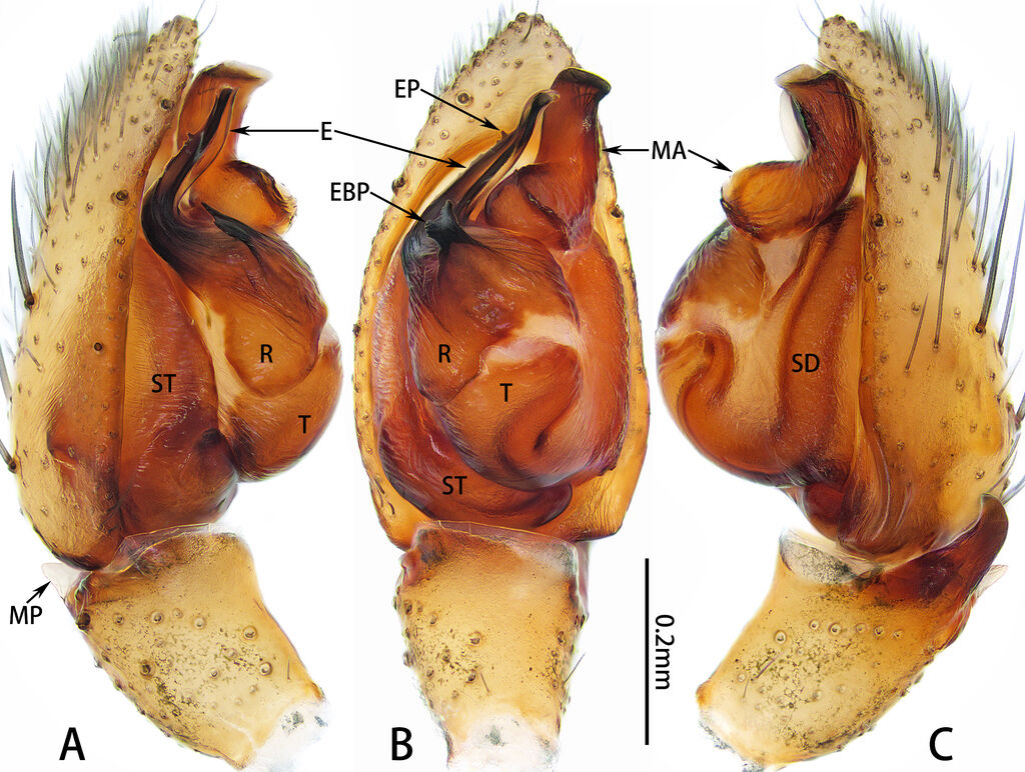
Male palp of *Xizangianaplankton* sp. nov. **A** prolateral view; **B** ventral view; **C** retrolateral view. Abbreviations: E = embolus, EBP = embolar base projection, EP= embolar process, MA = median apophysis, MP = membranous projection, R = embolar radix, SD = sperm duct, ST = subtegulum, T = tegulum.

**Figure 3.**
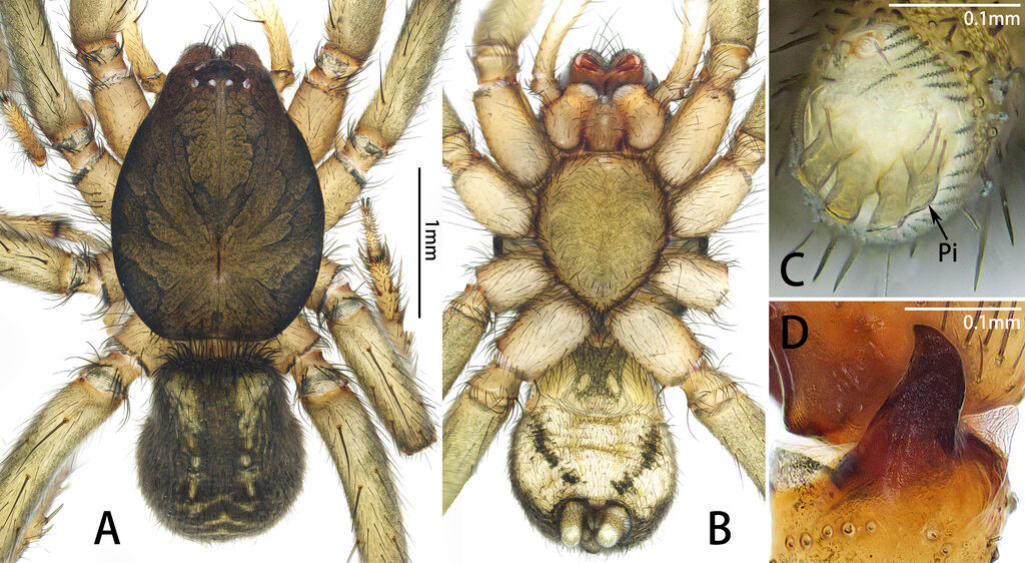
*Xizangianaplankton* sp. nov., male. **A, B** habitus, dorsal and ventral view; **C** spigots on anterior lateral spinneret; **D** retrolateral tibial apophysis, retrolateral view. Abbreviations: Pi = piriform gland spigots.

Palp (Figs 2, 3D). Femur and patella unmodified. Tibia with retrolateral apophysis, almost the length of tibia, slightly curved distally; and triangular membranous projection retro-dorsally. Cymbium pear-shaped, with length twice than width. Median apophysis twisted and wide at base, kidney-shaped in retrolateral view. Embolar radix relatively broad posteriorly. Embolar base with short, branched, distolateral projection. Embolus long and twisted, almost length of tegulum, with uneven prolateral edges and distal process.

**Figure 4.**
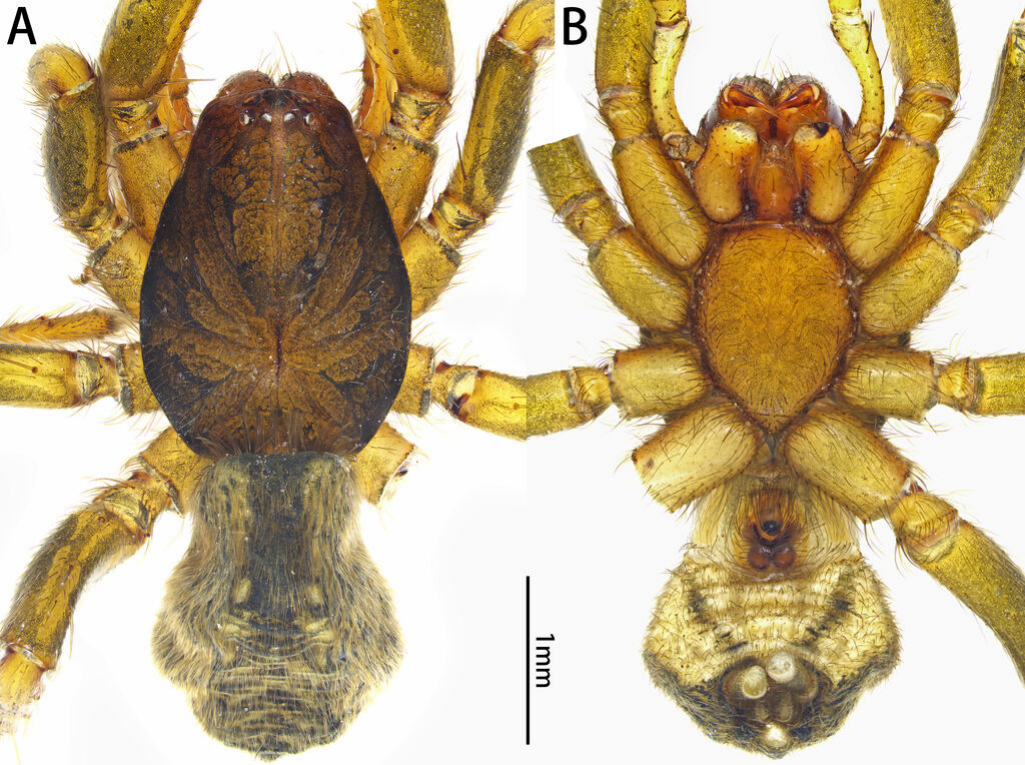
Habitus of *Xizangianaplankton* sp. nov., female. **A** dorsal view; **B** ventral view.

Female. Paratype: total length 3.99; carapace 2.20 long, 1.62 wide; abdomen 1.79 long, 1.50 wide. Eye sizes and interdistances: AME 0.07, ALE 0.12, PME 0.08, PLE 0.09; AME‒AME 0.05, AME‒ALE 0.01, PME‒PME 0.06, PME‒PLE 0.06, ALE‒PLE 0.05. Leg measurements: I 5.15 (1.46, 0.84, 1.24, 0.81, 0.80), II 4.49 (1.40, 0.68, 0.95, 0.73, 0.73), III 4.35 (1.18, 0.57, 0.74, 1.02, 0.84), IV 6.22 (1.74, 0.76, 1.21, 1.57, 0.94). Cheliceral promargin (Fig. 1A) and colour in alcohol (Fig. 4) as in male.

**Figure 5.**
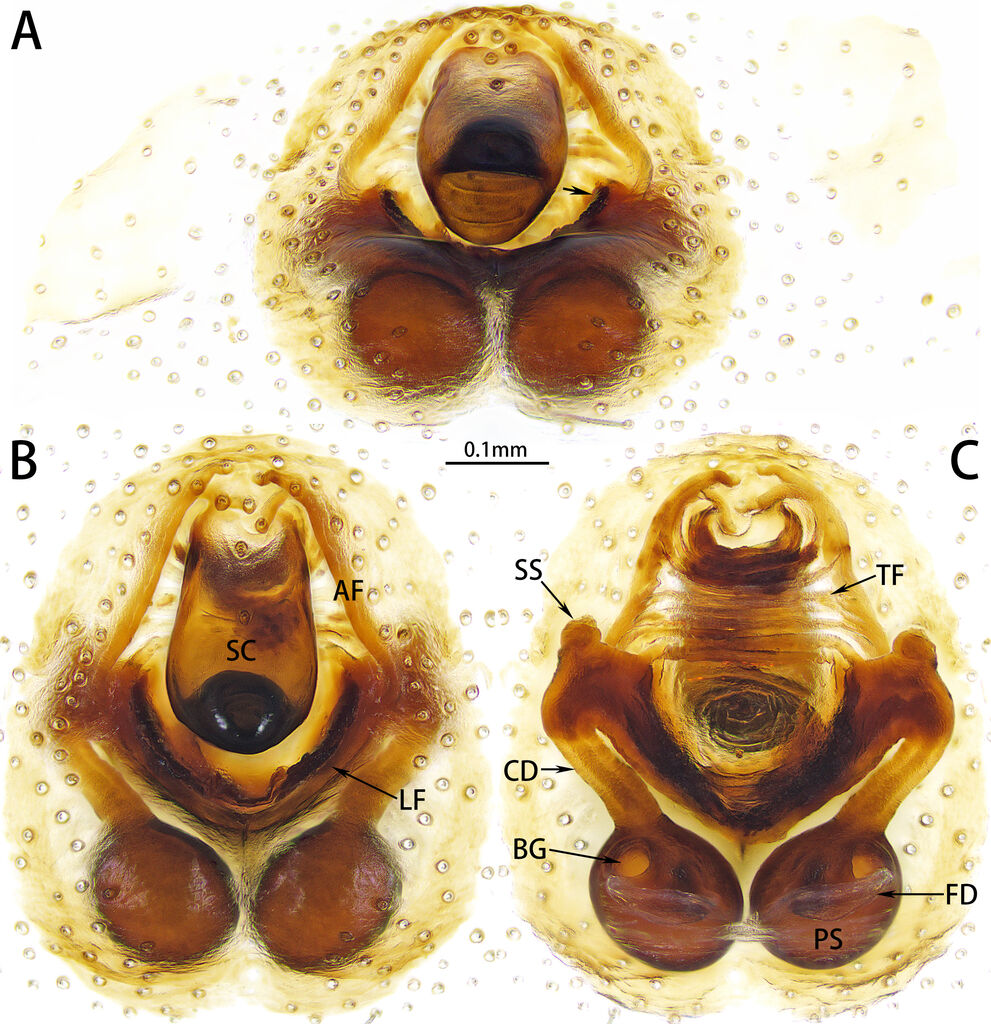
Epigyne of *Xizangianapalnkton* sp. nov. **A** oblique ventral view, with arrow pointing to copulatory opening; **B** ventral view; **C** dorsal view. Abbreviations: AF = anterior fold, BG = Bennett’s gland, CD = copulatory duct, FD = fertilisation ducts, LF = lateral fold, PS = primary spermathecae, SC = scape, SS = secondary spermathecae, TF = transversal folds.

Epigyne (Fig. 5). Epigynal plate elongated oval, with length/width ratio almost 6/5. Atrium almost elongate-diamond-shaped, with eight weakly-sclerotised transversal folds. Anterior fold well sclerotised. Scape long and wide, with a ratio of length to width more than 1.5, hollow inside, opening at the end. Lateral folds V-shaped, well sclerotised, approximately 4/5 width of anterior fold. Secondary spermathecae oval, small. Copulatory duct curved inwards. Primary spermathecae globular, large.

#### Diagnosis

Male resembles *X.rigaze* (Song, Zhu & Zhang, 2004) in palp structures, but can be distinguished by the relatively broad embolar base projection and posterior embolar radix; and the presence of a distal embolar process (Fig. 2; vs. the relatively narrow embolar base projection and posterior embolar radix; and the distal embolar process absent in *X.rigaze*, (see Liu et al. (2023): fig. 14; Song et al. (2004): figs. 143C–D). Female resembles *X.longlin* Liu & Zhang, 2023 in epigyne structures, but can be distinguished by the almost elongate-diamond-shaped atrium; the relatively broad lateral folds, approximately 4/5 width of anterior fold; and the copulatory duct curved inwards (Fig. 5; vs. the almost trapezoidal atrium; the relatively narrow lateral folds, with approximately 1/2 width of anterior fold; and the copulatory duct curved outwards in *X.longlin*, see Liu et al. (2023): fig. 17).

#### Etymology

The species is named after Sheldon J. Plankton, the main character in SpongeBob SquarePants, as the scape and anterior fold of epigyne of this new species resemble the body and flagellum of Plankton; noun (name) in apposition.

#### Distribution

China (Xizang).

## Supplementary Material

XML Treatment for Xizangiana plankton
